# Neuroprotective Effects of *Lactobacillus plantarum* PS128 in a Mouse Model of Parkinson’s Disease: The Role of Gut Microbiota and MicroRNAs

**DOI:** 10.3390/ijms24076794

**Published:** 2023-04-05

**Authors:** Yan Zhang Lee, Shih-Hsuan Cheng, Min-Yu Chang, Yu-Fen Lin, Chien-Chen Wu, Ying-Chieh Tsai

**Affiliations:** 1 Biomedical Industry Ph.D. Program, National Yang Ming Chiao Tung University, Taipei 11221, Taiwan; 2 Bened Biomedical Co., Ltd., Taipei 10448, Taiwan; 3 Institute of Biochemistry and Molecular Biology, National Yang Ming Chiao Tung University, Taipei 11221, Taiwan

**Keywords:** Parkinson’s disease, microRNAs, gut dysbiosis, *Lactobacillus plantarum* PS128, suppressor of cytokine signaling 1

## Abstract

Parkinson’s disease (PD) is a neurodegenerative disease characterized by motor deficits and marked neuroinflammation in various brain regions. The pathophysiology of PD is complex and mounting evidence has suggested an association with the dysregulation of microRNAs (miRNAs) and gut dysbiosis. Using a rotenone-induced PD mouse model, we observed that administration of *Lactobacillus plantarum* PS128 (PS128) significantly improved motor deficits in PD-like mice, accompanied by an increased level of dopamine, reduced dopaminergic neuron loss, reduced microglial activation, reduced levels of inflammatory factors, and enhanced expression of neurotrophic factor in the brain. Notably, the inflammation-related expression of miR-155-5p was significantly upregulated in the proximal colon, midbrain, and striatum of PD-like mice. PS128 reduced the level of miR-155-5p, whereas it increased the expression of suppressor of cytokine signaling 1 (SOCS1), a direct target of miR-155-5p and a critical inhibitor of the inflammatory response in the brain. Alteration of the fecal microbiota in PD-like mice was partially restored by PS128 administration. Among them, *Bifidobacterium*, *Ruminiclostridium*_6, *Bacteroides*, and *Alistipes* were statistically correlated with the improvement of rotenone-induced motor deficits and the expression of miR-155-5p and SOCS1. Our findings suggested that PS128 ameliorates motor deficits and exerts neuroprotective effects by regulating the gut microbiota and miR-155-5p/SOCS1 pathway in rotenone-induced PD-like mice.

## 1. Introduction

Parkinson’s disease (PD) is a neurodegenerative disease characterized by the loss of dopaminergic neurons in the substantia nigra (SN), leading to motor deficits with hypokinesia, which primarily affects people around the age of 60 [1]. Growing evidence suggest that neuroinflammation mediated by microglia—the resident macrophage-like immune cells of the central nervous system—is critical in the pathogenesis of PD [2]. Microglia display different activation states that regulate their cellular functions [3]. For instance, the typical inflammatory type promotes inflammatory responses. Conversely, when microglia are activated in the immunosuppressive state, they secrete anti-inflammatory cytokines and trophic molecules that promote repair and restore homeostasis [3]. Bartels et al. reported that microglia in the classical activation state—known as M1 microglia—are significantly active in the SN of PD-like mice, secreting inflammatory factors such as tumor necrosis factor-alpha (TNF-α), and interleukin-1 beta (IL-1β), thus leading to the degeneration of dopaminergic neurons [4].

MicroRNAs (miRNAs) are small noncoding RNAs that are crucial regulators of the gene expression involved in immunity and inflammation [5,6]. PD-related genes and genes involved in neuroinflammation are regulated by miRNAs and hence might contribute to the pathogenesis of PD [7]. In addition, miRNAs influence the progression of PD by modulating the microglial polarization state [6,8]. The altered expression of miRNAs, including that of miR-124 and miR-155-5p, contributes to PD progression through microglial activation and polarization processes [9,10]. Gut microbiota dysregulation has also been reported in PD, and several studies have reported a relationship between gut microbiota composition and miRNAs. In particular, Du et al. observed that the expression of miR-146a was induced by infection with *Listeria monocytogenes*, with miR-146a-deficient mice having a differentially developed gut microbiome and being more resistant to infection with *L. monocytogenes* compared with wild-type mice [11].

Substantial evidence has supported the notion that gut microbiota constitute a vital regulator of PD [12]. For instance, patients with PD often exhibit intestinal inflammation and digestive tract abnormalities many years before motility disorders [13]. A study in 2016 reported that mice receiving fecal microbes from patients with PD displayed significant defects in motility compared with mice that received microbiota from healthy controls [14]. Recently, many studies have reported that probiotics benefit PD populations by improving intestinal inflammation and neuroinflammation, which inhibit the subsequent loss of dopaminergic neurons via the microbiota–gut–brain axis (MGBA) [15,16,17]. Some strains of *Lactobacillus* and *Bifidobacterium* have the potential to inhibit the overgrowth of pathogenic bacteria, such as *Escherichia coli* and *Klebsiella pneumoniae*, the abundance of which are increased in patients with PD, leading to gut microbiota dysbiosis and inflammation [17,18]. Administration of polymannuronic acid combined with *Lactobacillus rhamnosus* GG reportedly improves the integrity of the blood–brain barrier and increases the expression of brain-derived neurotrophic factor (BDNF) and glial cell-derived neurotrophic factor, thereby inhibiting apoptosis of striatal cells in a PD mouse model [19]. Furthermore, probiotics interfere with the MGBA balance and influence the expression of miRNAs [20,21]. For instance, the probiotic *E. coli* strain Nissle 1917 regulated the expression of miRNAs involved in the inflammatory response in colitic mice [22]. Another study reported that *Lactobacillus plantarum* Z01 induced the expression of miRNAs that participated in the alleviation of cecal inflammation [23]. Therefore, the ability of probiotics to regulate the expression of miRNAs is important for maintaining the balance of the intestinal microenvironment.

Psychobiotics are defined as probiotics that when ingested in adequate quantities confer mental health benefits to the host [24]. *Lactobacillus plantarum* PS128 (PS128)—a novel psychobiotic strain—reportedly improves tic-like behavior and stabilizes dopamine transmission in a 2,5-dimethoxy-4-iodoamphetamine-induced hyperactivity rat model by modulating MGBA [25]. A pilot study indicated that after 12 weeks of PS128 supplementation, patients with PD exhibited remarkable improvements in Unified Parkinson’s Disease Rating Scale (UPDRS) motor scores and quality of life, suggesting that PS128 can be considered as an adjunctive agent in PD treatment [26]. In our previous studies, PS128 significantly improved motor function, diminished nigrostriatal dopaminergic neuronal cell death, and enhanced the levels of neurotransmitters in acute models of PD induced by 6-hydroxydopamine (6-OHDA) and 1-methyl-4-phenyl-1,2,3,6-tetrahydropyridine (MPTP) [27,28]. Similar to 6-OHDA and MPTP, rotenone is commonly used to induce symptoms of PD in rodents. MPTP and 6-OHDA cause extensive loss of dopaminergic neurons, leading to motor deficits but with no α-synuclein aggregation in rodents; in contrast, treatment with rotenone causes dopaminergic neurodegeneration, resulting in motor deficits and aggregation of α-synuclein [29].

Therefore, we aimed to investigate the neuroprotective effects of PS128 in a chronic rotenone-induced experimental mouse model of PD. We further evaluated its effect on the levels of miRNAs expression and gut microbiota composition. Our recent data demonstrated that PS128 ameliorates motor deficits and exerts neuroprotective properties by regulating gut microbiota and the miR-155-5p/SOCS1 pathway in rotenone-induced PD-like mice. We believe our findings will provide comprehensive insights into the effects of PS128 on PD.

## 2. Results

### 2.1. L. plantarum PS128 Improved Motor Deficits and Increased Dopamine Level in the Striatum of Rotenone-Induced PD-like Mice

**Figure 1:**
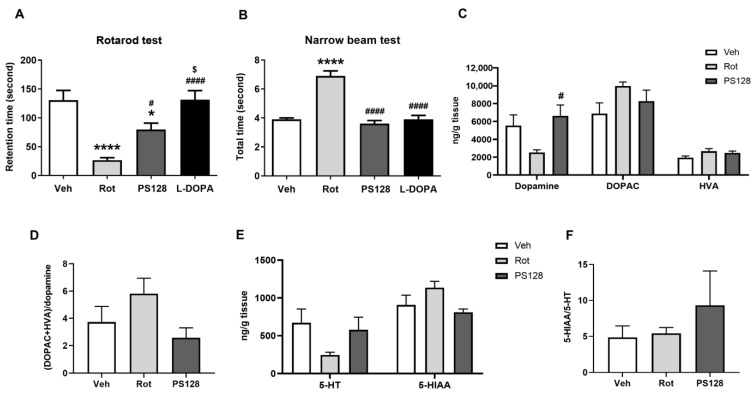
Effects of *L. plantarum* PS128 on motor deficits and neurotransmitter levels in rotenone-induced PD-like mice. (**A**) Total time spent on the rotarod. (**B**) Total walking time on a narrow beam. (**C**) Levels of striatal dopamine and its metabolites—3,4-dihydroxyphenylacetic acid (DOPAC) and homovanillic acid (HVA). (**D**) The ratio of dopamine turnover. (**E**) Levels of striatal serotonin (5-hydroxytryptamine; 5-HT) and its metabolite—5-hydroxyindoleacetic acid (5-HIAA). (**F**) The ratio of 5-HT turnover. N = 10 per group, * *p* < 0.05, **** *p* < 0.0001 compared with the Veh group; ^#^ *p* < 0.05, ^####^ *p* < 0.0001 compared with the Rot group; ^$^ *p* < 0.05 compared with the PS128 group.

We assessed the potential neuroprotective effects of PS128 in rotenone-induced PD-like mice using rotarod and narrow beam tests. We observed that the rotenone (Rot) group exhibited a significant decrease in rotarod latency and an increase in total walking time in the narrow beam test compared with those in the vehicle (Veh) group (Figure 1A,B; *p* < 0.0001). Conversely, PS128 administration significantly increased the rotarod retention time (*p* < 0.05) and decreased the total time spent on the narrow beam (*p* < 0.0001) compared to those with the Rot group (Figure 1A,B). Clinically, 3,4-dihydroxy-L-phenylalanine (L-DOPA) is a common drug used to treat motor symptoms in PD and was thus used as an effective behavioral test control in the present study. We observed that L-DOPA significantly attenuated rotenone-induced motor deficits in the rotarod and narrow beam tests compared with those in the Rot group (Figure 1A,B; *p* < 0.0001). The effect of L-DOPA, the positive control, on improving retention time in the rotarod test was slightly better than PS128, showing a 1.64-fold increase. However, in terms of reducing the total time in the narrow beam test, both L-DOPA and PS128 had similar effects, with only a 1.08-fold increase observed in the L-DOPA group compared to the PS128 group. Subsequently, we analyzed the effects of PS128 on the monoamine neurotransmitters, dopamine and serotonin (5-hydroxytryptamine; 5-HT), and their metabolites—3,4-dihydroxyphenylacetic acid (DOPAC), homovanillic acid (HVA), and 5-hydroxyindoleacetic acid (5-HIAA)—in the striatum of rotenone-treated mice. We observed that rotenone treatment did not significantly impact dopamine, DOPAC, HVA, 5-HT, and 5-HIAA levels compared with those in the Veh group (Figure 1C,E). However, PS128 significantly increased the level of dopamine compared with that in the Rot group (Figure 1C; *p* < 0.05). An increase in dopamine turnover has been reported as a compensatory mechanism for the loss of dopaminergic neurons in PD [30]. Furthermore, 5-HT dysregulation leads to both motor and nonmotor symptoms, such as tremors and depression [31]. Hence, we examined dopamine turnover ((DOPAC + HVA)/dopamine) and 5-HT turnover (5-HIAA/5-HT) among experimental groups but did not detect any significant differences (Figure 1D,F).

### 2.2. L. plantarum PS128 Attenuated Rotenone-Induced Reduction in the Numbers of Tyrosine Hydroxylase-Positive (TH^+^) Dopaminergic Neurons in the SN and Striatum

**Figure 2:**
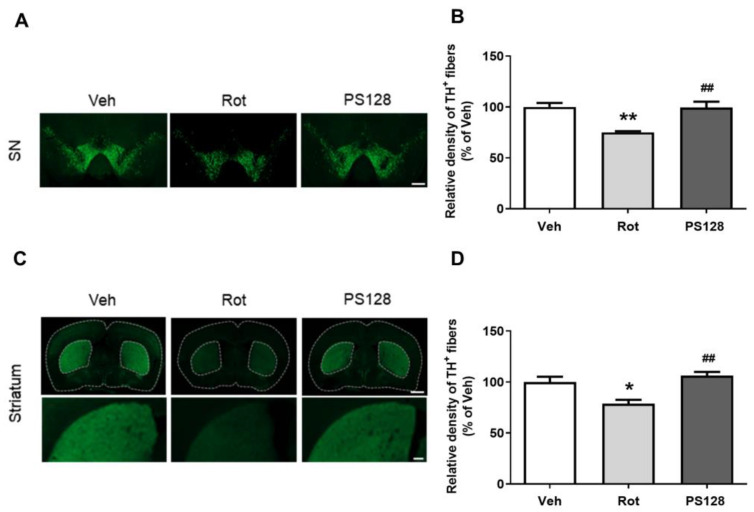
*L. plantarum* PS128 attenuated the reduction of tyrosine hydroxylase-positive (TH^+^) dopaminergic neurons in rotenone-induced PD-like mice. (**A**,**C**) Representative images of TH immunostaining in the substantia nigra (SN) and striatum of mice, respectively. (**B**,**D**) Quantitative analysis of the density of TH^+^ immunostaining in the SN and striatum of mice, respectively. N = 5 per group, * *p* < 0.05, ** *p* < 0.01 compared with the Veh group; ^##^ *p* < 0.01 compared with the Rot group. Scale bar = 500 μm in (**A**); scale bar = 1 mm and 200 μm on the top and bottom panel of (**C**), respectively.

We further investigated the effects of PS128 on the survival of dopaminergic neuronal cells in the SN and striatum. Immunofluorescence staining revealed a 30% and 25% reduction in the number of TH^+^ dopaminergic neurons in the SN (Figure 2A,B; *p* < 0.01) and striatum (Figure 2C,D; *p* < 0.05), respectively, in the Rot group compared with those in the Veh group. Conversely, we observed that administration of PS128 significantly rescued the rotenone-induced reduction in the number of TH^+^ cells in the SN and striatum compared with those in the Rot group (Figure 2A–D; *p* < 0.01). Thus, PS128 exerted beneficial effects by restoring TH-expressing neurons in rotenone-induced PD-like mice.

### 2.3. L. plantarum PS128 Reduced Rotenone-Induced Microglial Activation and Increased the Level of Brain Neurotrophic Factor in the Midbrain

**Figure 3:**
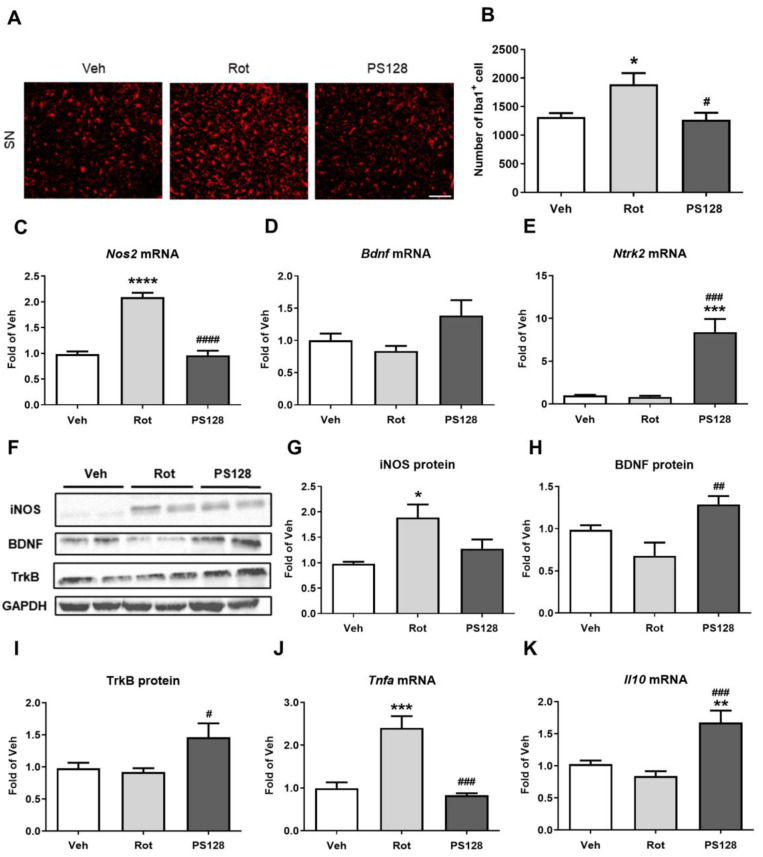
Effects of *L. plantarum* PS128 on rotenone-induced microglial activation, neurotrophic factor, and inflammation in the brain. (**A**) Representative images of ionized calcium-binding adaptor molecule-1 (Iba1) immunostaining in the SN. (**B**) Quantitative analysis of Iba1^+^ cells in the SN of mice. (**C**–**E**) The levels of gene expression of nitric oxide synthase 2 (*Nos2*), brain-derived neurotrophic factor (*Bdnf*), and neurotrophic receptor tyrosine kinase 2 (*Ntrk2*) in the midbrain. (**F**) Representative blots of inducible nitric oxide synthase (iNOS), BDNF, and tropomyosin receptor kinase B (TrkB) in the midbrain. (**G**–**I**) Quantitative analysis of iNOS, BDNF, and TrkB blots. (**J**,**K**) The gene expression levels of tumor necrosis factor-alpha (*Tnfa*) and interleukin-10 (*Il10*). N = 5 per group, * *p* < 0.05, ** *p* < 0.01, *** *p* < 0.001, **** *p* < 0.0001 compared with the Veh group; ^#^ *p* < 0.05, ^##^ *p* < 0.01, ^###^ *p* < 0.001, ^####^ *p* < 0.0001 compared with the Rot group. Scale bar = 100 μm in (**A**).

To evaluate the effects of PS128 on rotenone-induced microglial activation in the SN, we performed immunofluorescence staining against ionized calcium-binding adaptor molecule-1 (Iba1), which is strongly upregulated in activated microglia. We detected that the expression of Iba1 was increased by 40% in the SN of the Rot group compared with that in the Veh group (Figure 3A,B; *p* < 0.05). However, administration of PS128 significantly reduced the rotenone-induced expression of Iba1 in the SN of mice (Figure 3A,B; *p* < 0.05). Furthermore, we observed that the expression of inducible nitric oxide synthase (iNOS; also known as nitric oxide synthase 2; *Nos2*)—a microglia-mediated inflammation factor—was significantly increased in the Rot group compared with that in the Veh group (Figure 3C; *p* < 0.0001, Figure 3F,G; *p* < 0.05). The presence of rotenone increased the expression of *Nos2* mRNA and iNOS protein by 2.13-fold and 1.92-fold, respectively. The expression of *Nos2* mRNA and iNOS protein in the PS128 group was similar to that of the Veh group, with a 0.98-fold and 1.3-fold change, respectively. PS128 administration significantly decreased the mRNA level of *Nos2* (Figure 3C; *p* < 0.0001), and we further noticed a decreasing trend in the level of iNOS protein in the PS128 group compared with those in the Rot group (Figure 3G, *p* = 0.0797). We next examined the expression of BDNF and its receptor—tropomyosin receptor kinase B (TrkB; also known as neurotrophic receptor tyrosine kinase 2; *Ntrk2*)—in the midbrain. The Rot group showed similar expression levels of *Bdnf* mRNA, *Ntrk2* mRNA, BDNF protein, and TrkB protein to those of the Veh group, with respective fold changes of 0.84, 0.81, 0.69, and 0.94. Compared to the Veh group, the expression levels of *Bdnf* mRNA, *Ntrk2* mRNA, BDNF protein, and TrkB protein were higher in the PS128 group, with fold changes of 1.38, 8.40, 1.31, and 1.49, respectively. PS128 administration slightly increased the expression of *Bdnf* (Figure 3D; *p* = 0.0689) and significantly elevated the level of *Ntrk2* mRNA (Figure 3E; *p* < 0.001) compared with those in the Rot group. We also observed that the protein levels of BDNF and TrkB were significantly increased (Figure 3F,H; *p* < 0.01, I; *p* < 0.05, respectively) in the PS128 group compared with those in the Rot group. These results illustrated the neuroprotective effects of PS128 in reducing microglial activation and increasing the neurotrophin level in the brain.

### 2.4. L. plantarum PS128 Alleviated Rotenone-Induced Neuroinflammation and Promoted Anti-Inflammatory Effects in Brain Tissue

We measured the expression levels of the proinflammatory cytokine TNF-α and that of the anti-inflammatory cytokine interleukin-10 (IL-10) in the midbrain region containing the SN. We detected a significant 2.42-fold increase in the level of *Tnfa* mRNA in the Rot group, and a 0.84-fold lower expression of *Tnfa* mRNA in the PS128 group compared with those in the Veh group (Figure 3J; *p* < 0.001). In contrast, PS128 administration significantly reduced the level of *Tnfa* mRNA in rotenone-induced PD-like mice (Figure 3J; *p* < 0.001). Additionally, we observed that the expression of *Il10* mRNA was significantly higher in the PS128 group than in the Veh and Rot groups by 1.63-fold and 1.99-fold, respectively (Figure 3K; *p* < 0.01, *p* < 0.001, respectively). However, we did not detect any significant differences in the level of *Il10* mRNA between the Rot and Veh groups, with only a 0.82-fold decrease in the Rot group (Figure 3K).

### 2.5. Effects of L. plantarum PS128 on MiRNAs Expression in the Proximal Colon, Midbrain, and Striatum of Mice with Rotenone-Induced PD

As miRNAs play a crucial role in the development of PD, one of the aims of this study was to reveal the expression patterns of miRNAs in different tissues of rotenone-induced PD-like mice. Several well-studied miRNAs, which are related to neuroinflammation and PD, were selected based on previous studies [32,33]. As a result, we identified the expression of these miRNAs in the proximal colon, midbrain, and striatum of PD-like mice. We observed that the expression of miR-155-5p and miR-223-3p was significantly increased in the proximal colon in the Rot group (Table 1; *p* < 0.01, *p* < 0.05, respectively). However, the administration of PS128 significantly reduced the expression of both miR-155-5p and miR-223-3p (Table 1; *p* < 0.001 and *p* < 0.05, respectively). In addition, PS128 administration significantly increased the expression of miR-21-5p, miR-34a-5p, and miR-146a-5p compared with that in the Veh group (Table 1; *p* < 0.01, *p* < 0.05, and *p* < 0.01, respectively). Likewise, the expression of miR-34a-5p and miR-155-5p was significantly increased in the midbrain of the Rot group (Table 1; *p* < 0.05), whereas it was reduced in the PS128 group (Table 1; *p* < 0.05, *p* < 0.01, respectively). PS128 administration also decreased the expression of miR-223-3p compared to that in the Rot group (Table 1; *p* < 0.05). In the striatum, the expression of miR-155-5p was significantly increased in the Rot group (Table 1; *p* < 0.05), whereas it was decreased in the PS128 group (Table 1; *p* < 0.01). These results indicated that the expression of miR-155-5p was significantly increased by rotenone induction and reduced by PS128 administration; notably, this trend was consistent in all tested tissues.

### 2.6. L. plantarum PS128 Administration Upregulated Expression of Suppressor of Cytokine Signaling 1 (SOCS1) in Rotenone-Induced PD-Like Mice

**Figure 4:**
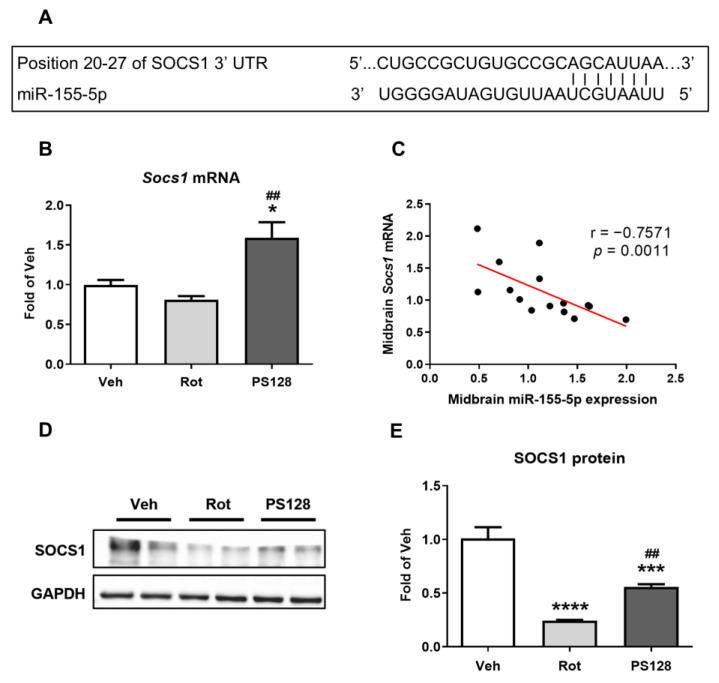
*L. plantarum* PS128 administration upregulated the expression of suppressor of cytokine signaling 1 (SOCS1) in rotenone-induced PD-like mice. (**A**) The conserved binding sites for miR-155-5p in the 3′ untranslated region (UTR) of SOCS1. (**B**) The gene expression level of *Socs1* in the midbrain. (**C**) Correlation between the expression of miR-155-5p and *Socs1* mRNA as determined by Spearman’s rank test. (**D**) Representative blots of SOCS1 in the midbrain. (**E**) Quantitative analysis of SOCS1 blots. N = 5 per group, * *p* < 0.05, *** *p* < 0.001, **** *p* < 0.0001 compared with the Veh group; ^##^ *p* < 0.01 compared with the Rot group.

We further explored the role of miR-155-5p in PD. According to previous studies, analysis using TargetScan revealed conserved binding sites for miR-155-5p in the 3′ untranslated region (UTR) of SOCS1; thus, SOCS1 is considered a direct target of miR-155-5p [34,35] (Figure 4A). We quantified the expression of SOCS1 in the midbrain and observed that PS128 significantly increased the level of *Socs1* mRNA compared with that in the Rot group (Figure 4B; *p* < 0.01). In addition, we detected a significant negative correlation between the expression of miR-155-5p and that of *Socs1* in the midbrain (Figure 4C; r = −0.7571, *p* = 0.0011). We also noticed that the protein level of SOCS1 was significantly reduced in the Rot group compared with that in the Veh group (Figure 4D, E; *p* < 0.0001), whereas there was an increase in the expression of SOCS1 in the PS128 group compared with that in the Rot group (Figure 4D,E; *p* < 0.01).

### 2.7. Rotenone Treatment and L. plantarum PS128 Altered Fecal Microbiota Profile

**Figure 5:**
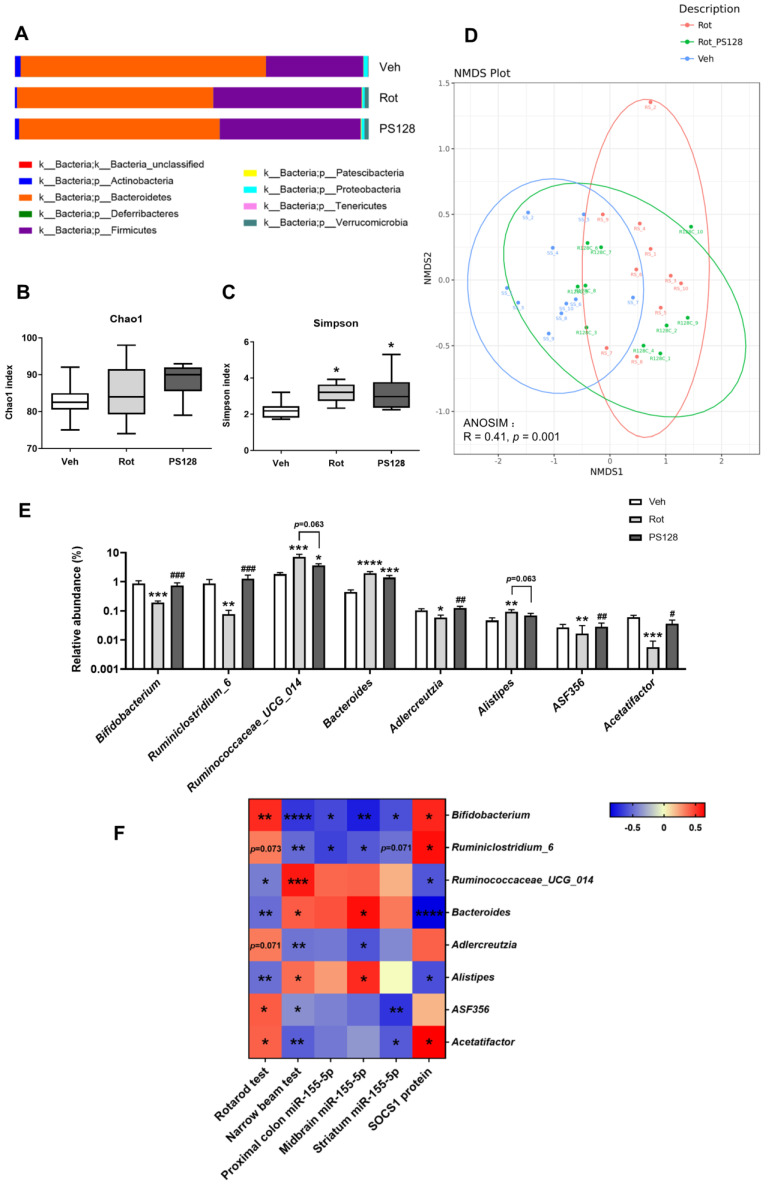
Effects of *L. plantarum* PS128 on the fecal microbiome in rotenone-induced PD-like mice. (**A**) Relative abundance of bacteria at the phylum level. Alpha diversity is indicated by Chao1 (**B**) and Simpson (**C**) diversity indices. (**D**) Beta diversity is indicated by nonmetric multidimensional scaling (NMDS). (**E**) Analysis of the relative abundance of bacteria at the genus level among all groups. N = 10 per group. (**F**) Heat map of the Spearman’s rank correlation test representing the correlation between bacteria at the genus level and behavioral performance, expression of miR-155-5p in different tissues, and midbrain expression of SOCS1. N = 10 per group in the correlation between bacteria with behavioral performance; n = 5 per group in the correlation between bacteria with tissue expression of miR-155-5p and midbrain expression of SOCS1. * *p* < 0.05, ** *p* < 0.01, *** *p* < 0.001, **** *p* < 0.0001 compared with the Veh group; ^#^ *p* < 0.05, ^##^ *p* < 0.01, ^###^ *p* < 0.001 compared with the Rot group.

We investigated the effect of rotenone treatment and PS128 administration on mouse gut microbiota. Briefly, we extracted DNA from mouse fecal samples and performed 16S rRNA microbiome analysis. Sequencing was performed with good quality (Supplementary Table S1). An analysis of the microbiota composition revealed that *Bacteroidetes* and *Firmicutes* were the dominant phyla among groups (Figure 5A). We assessed the alpha diversity of gut microbiota using the Chao1 and Simpson indices. We did not detect any significant differences between groups in the Chao1 index; however, rotenone treatment and PS128 administration significantly elevated the Simpson index, suggesting a major increase in the observed species diversity (Figure 5B,C; *p* < 0.05). Furthermore, we generated a nonmetric multidimensional scaling (NMDS) plot and analyzed it using analysis of similarities (ANOSIM) to evaluate the beta diversity. We observed a distinct clustering of the fecal microbiota composition between groups (Figure 5D; ANOSIM R = 0.41, *p* = 0.001). In addition to beta diversity, we also performed linear discriminant analysis effect size (LEfSe) analysis to identify differentially presented taxa (Supplementary Figure S1) and statistical analysis to determine differences in the relative abundance of microbes among groups. We accordingly observed that at the genus level, the relative abundance of *Bifidobacterium*, *Ruminiclostridium*_6, *Adlercreutzia*, ASF356, and *Acetatifactor* were decreased in the rotenone-treated group and enriched in the PS128 group (Figure 5E). In contrast, the abundance of *Ruminococcaceae*_UCG_014, *Bacteroides*, and *Alistipes* were significantly increased in the Rot group and decreased in the PS128 group (Figure 5E). These results demonstrated that rotenone treatment altered the composition of gut microbiota, which was restored and reshaped by PS128.

### 2.8. Correlation of PS128-Modulated Gut Bacteria and Behavioral Tests

We used Spearman correlation analysis to investigate the correlation between PS128-modulated gut bacteria and behavioral performance in PD-like mice (Figure 5F). We observed that the abundances of *Bifidobacterium*, ASF356, and *Acetatifactor* were positively correlated with the rotarod retention time, whereas those of *Ruminococcaceae*_UCG_014, *Bacteroides*, and *Alistipes* were negatively correlated with the rotarod retention time. We also observed a negative correlation between the abundance of *Bifidobacterium*, *Ruminiclostridium*_6, *Adlercreutzia*, ASF356, and *Acetatifactor* and time spent in the narrow beam test. In contrast, the abundances of *Ruminococcaceae*_UCG_014, *Bacteroides*, and *Alistipes* were positively correlated with the time spent in the narrow beam test. These results suggested that increased abundances of *Bifidobacterium*, *Ruminiclostridium*_6, *Adlercreutzia*, ASF356, and *Acetatifactor*, and decreased abundances of *Ruminococcaceae*_UCG_014, *Bacteroides*, and *Alistipes* were positively associated with improvement in rotenone-induced motor deficits.

### 2.9. Correlation of PS128-Modulated Gut Bacteria and Expression of Tissue MiR-155-5p and SOCS1

We further used Spearman’s correlation analysis to identify the association between PS128-modulated gut bacteria and the expression of miR-155-5p in the proximal colon, midbrain, and striatal tissues, and the expression of SOCS1 in the midbrain (Figure 5F). We observed that the abundances of *Bifidobacterium* and *Ruminiclostridium*_6 were negatively correlated with the levels of expression of miR-155-5p in proximal colon and midbrain tissues. However, in the striatum, only *Bifidobacterium* was negatively correlated with the expression of miR-155-5p. Furthermore, we detected a significant positive correlation between the abundance of *Bifidobacterium* and *Ruminiclostridium*_6 and the level of SOCS1 protein in the midbrain. In contrast, the abundance of *Bacteroides* and *Alistipes* were positively associated with the expression of miR-155-5p in the midbrain and negatively associated with that of SOCS1. Overall, these results indicated that the abundance of *Bifidobacterium*, *Ruminiclostridium*_6, *Bacteroides*, and *Alistipes* were correlated with the miR-155-5p/SOCS1 pathway in rotenone-induced PD-like mice.

## 3. Discussion

PD is a common neurodegenerative disease affecting older individuals that causes movement disorders and affects the quality of life [36]. Therefore, the development of novel strategies to restore locomotion in PD, including the intake of probiotics that has been reported to show beneficial effects, have been encouraged. In this study, we examined the possibility of using PS128 to improve locomotor activity in PD-like mice induced by chronic rotenone treatment and investigated the miRNAs profile and gut microbiota to explore the underlying mechanisms. Our results suggested that administration of PS128 significantly improved motor deficits, increased dopamine levels, and prevented the loss of dopaminergic neurons in the nigrostriatal pathway (Figure 1 and Figure 2), confirming our previous observations [28]. PS128 administration also prevented microglial activation by suppressing the expression of Iba1 and iNOS (Figure 3). iNOS is a vital synthase involved in microglial activation that also serves as a proinflammatory M1 polarization marker [37]. In addition, PS128 increased the expression of the neuroprotective factor BDNF and upregulated the expression of its receptor TrkB (Figure 3). BDNF is thought to affect the dopaminergic nervous system [38] and regulate neuroinflammatory responses through the BDNF-TrkB pathway, the upregulation of which has been reported to be beneficial in PD [39]. Finally, PS128 administration significantly reduced the expression of *Tnfa* and increased that of *Il10* in the brain of PD-like mice (Figure 3), indicating that PS128 plays a role in modulating neuroinflammation.

Age is one of the greatest risk factors in PD [40]. Dysregulation of miRNAs has been associated with many age-related diseases [41]. Based on previous studies, we selected a panel of miRNAs, including miR-19b-3p, miR-21-5p, miR-34a-5p, miR-135a-5p, miR-146a-5p, miR-155-5p, and miR-223-3p, which are involved in pro- or anti-inflammatory signaling and are dysregulated in the ageing process or PD pathology [32,33,42]. To better characterize the neuroprotective effects of PS128 in PD-like mice, we examined the expression of miRNAs in the proximal colon, midbrain, and striatum, as the proximal colon is a major attachment site for probiotics [43], and the nigrostriatal region plays a critical role in PD. We observed that the expression of miR-155-5p and miR-223-3p was significantly increased in the proximal colon of PD-like mice; conversely, administration of PS128 significantly decreased their expression (Table 1). Similar results were observed in a recent study that showed that administration of the probiotic *Saccharomyces boulardii* reduced the increased expression of miR-155-5p and miR-223-3p in a dextran sodium sulphate model of mouse colitis [44]. In addition, PS128 promoted the expression of miR-21-5p, miR-34a-5p, and miR-146a-5p in the proximal colon. Most studies on miR-21 have focused on apoptosis and neuroinflammation and have suggested that miR-21 protects against neuronal apoptosis [45,46]. For instance, miR-146a was reportedly downregulated in patients with PD [47]. In contrast, the expression of miR-34a-5p and miR-155-5p was upregulated in the midbrain of PD-like mice (Table 1). Additionally, we observed reduced levels of expression of miR-34a-5p, miR-155-5p, and miR-223-3p in the PS128 group in our study. Notably, miR-34a-5p is involved in neuronal differentiation and brain ageing [48,49] and is reportedly upregulated in patients with PD [50]. Finally, miR-155-5p was the only miRNA with increased expression and was conversely significantly reduced following administration of PS128 in the striatum of PD-like mice (Table 1). Our results revealed that the expression of miRNAs was inconsistent in different tissues; however, miR-155-5p was the only miRNA of which the levels were increased and then reduced by PS128 administration in all tested tissues of PD-like mice. Therefore, miR-155-5p was selected for further analysis. We next investigated the expression of SOCS1, a direct target of miR-155-5p, in the brain region. We observed that PS128 significantly increased the expression of SOCS1 in rotenone-induced PD-like mice (Figure 4). Intriguingly, Spearman correlation analysis indicated that the expression of miR-155-5p was negatively correlated with that of *Socs1* mRNA in the midbrain tissue (Figure 4), confirming previous findings that miR-155 is a negative regulator of SOCS1 [9]. Our results suggested that rotenone treatment altered the expression of miR-155 and SOCS1, and these disturbances could be partially restored by PS128.

In this regard, we suggested that the miR-155-5p/SOCS1 pathway might be involved in the rotenone-induced progression of PD-like symptoms. miR-155-5p is commonly described as an inflammatory-associated miRNA that is dysregulated in neurodegenerative diseases involving neuroinflammatory signaling and exacerbation of nerve damage [9,33]. Amplification of miR-155 is accompanied by increased levels of proinflammatory cytokines, including IL-1β and TNF-α [51]. Previous studies have demonstrated that miR-155-5p is involved in regulating microglial activation and polarization. Specifically, the overexpression of miR-155 enhanced the activity of M1-type microglia in a kainic acid-induced seizure mouse model [52]. Zheng et al. suggested that propofol suppressed the LPS-induced neuroinflammatory responses of microglia through the regulation of the miR-155/SOCS1 pathway [53]. SOCS1 is an important negative regulator of inflammation [54]. Treatment with resveratrol has been reported to attenuate inflammatory responses, possibly through the upregulation of SOCS1 in MPTP-treated mice [55]. Similarly, in this study, we observed increased levels of miR-155-5p and TNF-α and a decreased level of SOCS1 in PD-like mice, and these effects were reversed by PS128 administration. Overall, our results supported the hypothesis that PS128 ameliorates motor deficits, suppresses microglial polarization toward the M1 phenotype, and reduces microglial activation in rotenone-induced PD-like mice, possibly through regulation of the miR-155-5p/SOCS1 pathway.

Alterations in gut microbiota have often been observed in patients with PD. In the present study, rotenone treatment significantly altered the composition of gut microbiota in PD-like mice compared with control mice (Figure 5). We observed that especially at the genus level, the relative abundances of *Bifidobacterium*, *Ruminiclostridium*_6, *Adlercreutzia*, ASF356, and *Acetatifactor* were decreased in rotenone-induced PD-like mice but enriched significantly following PS128 administration. In contrast, the relative abundances of *Ruminococcaceae*_UCG_014, *Bacteroides*, and *Alistipes* were increased in rotenone-induced PD-like mice but decreased by PS128 supplementation. The alterations in the gut microbiota in our study were similar to those reported by Perez-Pardo et al. They observed a significant decrease in the relative abundance of *Bifidobacterium* and an increase in the relative abundance of *Ruminococcaceae* in the cecum of rotenone-induced mice [56]. In addition, Minato et al. reported that the worsening of PD symptoms was associated with a lower abundance of *Bifidobacterium* [57]. A previous study proposed that the administration of *Bifidobacterium breve* had beneficial effects in the MPTP-induced mouse model of PD [58]. Contrary to our findings, Bhattarai et al. found that rotenone treatment led to an increase in the relative abundance of *Bifidobacterium* and a decrease in the relative abundance of *Ruminococcaceae*_UCG-014 [59]. However, the results of studies on *Ruminococcaceae* in PD have been controversial and might be related to the disease duration of PD [60]. ASF356 and *Acetatifactor* belong to the *Lachnospiraceae* family and recent studies have revealed that the abundance of the *Lachnospiraceae* and *Ruminiclostridium* were decreased, whereas those of *Alistipes* and phylum *Bacteroidetes* were increased in MPTP-induced PD-like mice [61,62,63]. In 2020, Zhang et al. identified *Adlercreutzia*, a genus associated with anti-inflammatory properties, as one of the PD-associated bacterial taxa in patients with PD [64]. Interestingly, a recent study indicated that the abundance of *Bacteroides* was not only associated with the severity of motor symptoms as defined by UPDRS scores but was also positively associated with the level of TNF-α in patients with PD [65]. Moreover, the abundance of the *Alistipes* genus was higher in patients with PD with mild cognitive impairment, and *Alistipes* was negatively associated with cognitive ability [66].

In our study, PS128 significantly restored the rotenone-induced alterations in the composition of gut microbiota; therefore, we further investigated whether these specific microbial taxa might be involved in the pathological progression of PD. We first correlated these specific microbial taxa with the improvement in rotenone-induced motor deficits (Figure 5). An increase in the abundances of *Bifidobacterium*, *Ruminiclostridium*_6, *Adlercreutzia*, ASF356, and *Acetatifactor*, and a decrease in those of *Ruminococcaceae*_UCG_014, *Bacteroides*, and *Alistipes* were correlated with an improvement in motor deficits of PD-like mice. Notably, we observed changes in the levels of some inflammation-related miRNAs in different tissues. Among the tested miRNAs, the expression of inflammation-associated miR-155-5p was consistently enhanced in different tissues of PD-like mice, which in turn was significantly decreased by PS128 administration. SOCS1, a direct target of miR-155-5p, is known to regulate proinflammatory responses. Thus, we further aimed to correlate PS128-modulated gut bacteria with the expression of miR-155-5p and SOCS1 (Figure 5). Strikingly, we discovered that several PS128-modulated gut bacteria, including *Bifidobacterium*, *Ruminiclostridium*_6, *Bacteroides*, and *Alistipes*, were correlated with the expression of miR-155-5p and SOCS1, indicating that PS128 might influence the expression of miRNAs by partially modulating the composition of gut microbiota. Increasing evidence has shown the potential of probiotics to manipulate the gut microbiome and regulate target genes through miRNAs to maintain host homeostasis, resulting in preventive and therapeutic effects [67]. However, whether changes in the levels of miRNAs are a cause or consequence of alterations in the composition of gut microbiota and the mechanism by which they affect the progression of PD requires further studies.

Although our study found significant effects of PS128 on PD-like mice, it is important to note that one limitation is that we did not explore the effect of PS128 on healthy mice. Since motor deficits are major symptoms of PD, it was difficult to study the influence of PS128 on control mice whose movements were normal. In our previous study using a MPTP-induced mouse model of PD, we found that control mice treated with saline and PS128 performed similarly in behavioral tests, including the rotarod and narrow beam tests [28]. Furthermore, the administration of PS128 in control mice did not show impacts on nigrostriatal dopaminergic neuron survival, levels of striatal monoamines and their metabolites, and neuroinflammation in a previous study [28]. In other words, the effects of PS128 were only apparent when PD-like symptoms were induced. Additionally, we discovered that although PS128 significantly increased the expression levels of dopamine, the neurotrophic factor BDNF and its receptor *Ntrk2* mRNA and TrkB, and anti-inflammatory cytokine *Il10* mRNA compared to the Rot group, the expression of these factors did not differ between Rot and Veh groups. Due to the fact that supplementation with PS128 significantly improved the rotenone-induced motor deficits as evaluated by rotarod and narrow beam tests in PD-like mice, we suggest that these factors may not be the primary mechanisms underlying the amelioration of PD-like symptoms, but their possibility in ameliorating PD-like symptoms cannot be ruled out and requires further investigation.

Overall, our study supported that the beneficial properties of PS128 might be attributed to the restoration of the composition of gut microbiota and the miR-155-5p/SOCS1 pathway. Our study also provided another perspective on using probiotics for the treatment or adjuvant therapy of PD. Moreover, changes in the expression of miRNAs could also be used as diagnostic markers or for monitoring treatment response in PD.

## 4. Materials and Methods

### 4.1. Preparation of L. plantarum PS128

In this study, we used *L. plantarum*—recently reclassified as *Lactiplantibacillus plantarum* [68]—PS128 lyophilized powder that was prepared by Centro Sperimentale Del Latte srl (CSL, Milan, Italy). Before oral administration, the PS128 powder was weighed and suspended in phosphate-buffered saline (PBS) to obtain a final concentration of 10^10^ colony-forming units (CFU)/mL.

### 4.2. Animal Treatment

Eight-week-old male C57BL/6J mice were purchased and housed under standard laboratory conditions. All animal procedures were approved by the Institutional Animal Care and Use Committee of the National Yang Ming Chiao Tung University (protocol number 1101208). Mice were randomly assigned to 4 groups (n = 10 in each group): vehicle (Veh), rotenone (Rot), PS128 (PS128), and L-DOPA (L-DOPA) groups. The Veh group only received an oral gavage of PBS (week 1–6). The Rot group received oral gavage of PBS (week 1–6) and rotenone. The PS128 group received oral gavage of PS128 (10^9^ CFU/d) (week 1–6) and rotenone. The L-DOPA group received oral gavage of PBS (D1–37), rotenone, L-DOPA, and benserazide hydrochloride (D38–44). To induce PD-like symptoms, the Rot, PS128, and L-DOPA groups were injected intraperitoneally with rotenone (2.5 mg/kg/d) for four consecutive weeks (weeks 3–6) [69]. Behavioral tests, including the rotarod and narrow beam tests, were conducted on days 43 and 44 of the experiment. At the end of the experiment, all mice were sacrificed for subsequent analysis.

### 4.3. Rotarod Test

A rotarod treadmill (RT-01, SINGA Technology Corporation, Taipei, Taiwan) was used to assess motor coordination in mice. Animals underwent three training sessions (1 per week) before the actual tests. For each training, mice were trained on the rotarod at constant speeds of 10, 15, and 20 rpm at least three times for a maximum of 3 min/time. Tests were performed on day 43 of the experiment with three trials at a constant speed of 30 rpm for 3 min. The retention time of mice was recorded during the observation period, and the average retention time was considered the final result.

### 4.4. Narrow Beam Test

A narrow beam test was performed to assess motor coordination and balance in mice. The narrow beam apparatus consisted of a wooden beam (50 cm long and 0.8 cm wide) and a black box at one end as the finish point [28]. The beam was elevated to 50 cm above the ground. During the training session, mice were placed in the black box for 5 min for adaptation. Then, mice were placed 10, 20, and 30 cm apart from the box and were trained to walk toward the box at least thrice for each length. Mice were trained three times in total, one time per week, for three consecutive weeks. During the tests, mice were placed at the end of the beam, facing the black box. The total time required to reach the black box was recorded, and the average time of the three trials was considered the final result.

### 4.5. Quantification of Monoamine Neurotransmitters and Their Metabolites

Monoamine neurotransmitters, dopamine and 5-HT, and their metabolites—DOPAC, HVA, and 5-HIAA—were detected using previously published high performance liquid chromatography–electrochemical detection methods [28]. Briefly, the striata of mice were weighed and lysed by sonication (4.5 m/s) in perchloric acid buffer. Samples were then centrifuged at 18,000× *g* for 5 min. Supernatants were collected, kept on ice for 30 min, and centrifuged at 18,000× *g* for 20 min. Supernatants were then transferred and filtered through a 0.22 μm polyvinylidene difluoride (PVDF) membrane (Merck Millipore, Darmstadt, Germany). The mobile phase was pumped at a constant flow rate of 0.2 mL/min to quantify dopamine, 5-HT, DOPAC, HVA, and 5-HIAA. Filtered samples were injected into the chromatographic system, and the concentrations of neurotransmitters and metabolites were interpolated using the standard curve.

### 4.6. Immunofluorescence

Mice were deeply anesthetized and perfused with 10% formalin fixative (JT Baker, Center Valley, PA, USA). Brains were removed, postfixed with 10% formalin for 24 h at 4 °C, and dehydrated thrice with 30% sucrose solution. Brains were sliced into 20 μm thick sections using a CryoStar NX70 Cryostat (Thermo Fisher Scientific, Vienna, Austria). Subsequently, brain sections were washed in PBS (pH 7.4), continued with antigen retrieval in citrate buffer (pH 6) for 15 min at 85 °C, and followed by 30 min incubation in PBS containing 0.25% Triton X-100 (PBST). Brain sections were then blocked with 0.5% bovine serum albumin (A2153, Sigma, St. Louis, MO, USA) for 1 h and incubated overnight with primary antibodies, rabbit anti-TH (1:500, AB152, Merck Millipore) or rabbit anti-Iba1 (1:500, GTX100042, GeneTex, Irvine, CA, USA), at 4 °C. Subsequently, sections were washed with PBST and incubated with secondary antibodies, goat anti-rabbit conjugated to green fluorescent Alexa Fluor 488 (1:1000, A-11034, Invitrogen, Carlsbad, CA, USA) or goat anti-rabbit conjugated to red fluorescent Alexa Fluor 594 (1:1000, A-11037, Invitrogen), for 2 h. Sections were washed with PBST and stained with DAPI (1:1000, D1306, Invitrogen). Slices were covered with Fluoromount-G™ Mounting Medium (00-4958-02; Invitrogen) and stored at 4 °C. Fluorescent signals were detected using a fluorescence microscope (BX63, Olympus, Tokyo, Japan), and images were analyzed using the ImageJ software.

### 4.7. Real-Time PCR Analysis

For mRNA detection, total RNA was extracted from 10–20 μg of tissue using the RNeasy Mini Kit (Qiagen, Hilden, Germany). The Reverse RevertAid First Strand cDNA Synthesis Kit (Thermo Fisher Scientific) was used for reverse transcription (RT). The mRNA levels were quantified using the KAPA SYBR^®^ FAST qPCR kit (Merck Millipore) on a QuantStudio™ 3 Real-Time PCR System (Thermo Fisher Scientific). The reaction conditions were as follows: 95 °C for 10 min, followed by 40 cycles of 95 °C for 15 s, and 60 °C for 30 s. Primer sequences were as follows: glyceraldehyde-3-phosphate dehydrogenase (*Gapdh*) forward, 5′-CAATGTGTCCGTCGTGGATCT-3′ and reverse, 5′-GTCCTCAGTGTAGCCCAAGATG-3′; *Nos2* forward, 5′-ACATCGACCCGTCCACAGTAT-3′ and reverse, 5′-CAGAGGGGTAGGCTTGTCTC-3′; *Bdnf* forward, 5′-CAAAAGGCCAACTGAAGC-3′ and reverse, 5′-CGCCAGCCAATTCTCTTT-3′; *Ntrk2* forward, 5′-CCACGGATGTTGCTGACCAAAG-3′ and reverse, 5′-GCCAAACTTGGAATGTCTCGCC-3′; *Tnfa* forward, 5′-ATGAGCACAGAAAGCATGATC-3′ and reverse, 5′-TACAGGCTTGTCACTCGAATT-3′; *Il10* forward, 5′-ATGCTGCCTGCTCTTACTGACTG-3′ and reverse, 5′-CCCAAGTAACCCTTAAAGTCCTGC-3′; and *Socs1* forward, 5′-TGGGCACCTTCTTGGTGCGC-3′ and reverse, 5′-GGCAGTCGAAGGTCTCGCGG-3′. The relative levels of mRNA expression were normalized to those of *Gapdh*.

For miRNA detection, we referred to a previous study [70]. In brief, total RNA, including miRNA, was extracted from 10–20 μg of tissue using the miRNeasy Mini Kit (Qiagen). RT was performed using the miScript II RT Kit (Qiagen), and the miScript SYBR Green PCR Kit (Qiagen) was used to quantify miRNAs expression according to the manufacturer’s instructions. Reaction conditions were as follows: 95 °C for 15 min, followed by 40 cycles of 94 °C for 15 s, 55 °C for 30 s, and 70 °C for 30 s. MiScript Primer Assays (Qiagen) specific for miR-19b-3p (MIMAT0000513), miR-21-5p (MIMAT0000530), miR-34a-5p (MIMAT0000542), miR-135a-5p (MIMAT0000147), miR-146a-5p (MIMAT0000158), miR-155-5p (MIMAT0000165), and miR-223-3p (MIMAT0000665) were used, and RNU6B (U6) was used as the reference gene. MiRNAs expression was calculated using the delta threshold cycle method and were normalized to that of U6.

### 4.8. Western Blot Analysis

For Western blot analysis, midbrain tissues were collected to detect the levels of iNOS, BDNF, TrkB, and SOCS1. Tissue samples were homogenized in radio immuno precipitation assay buffer with protease inhibitor (Merck Millipore). Samples were centrifuged at 12,000× *g* to collect supernatants. The protein concentration in the supernatants was determined using a Bradford protein assay kit (Bio-Rad, Hercules, CA, USA). Equal amounts of protein were separated using 10% or 12.5% SDS-PAGE and transferred onto PVDF membranes (Roche Diagnostics, Laval, QC, Canada). PVDF membranes were blocked with 5% nonfat milk for 1 h at room temperature. Subsequently, PVDF membranes containing target proteins were incubated with respective primary antibodies: iNOS (1:1000, 13120S, Cell Signaling Technology, Danvers, MA, USA), BDNF (1:1000, GTX132621, GeneTex), TrkB (1:1000, ab187041, Abcam, Cambridge, UK), SOCS1 (1:1000, 3950T, Cell Signaling Technology), and GAPDH (1:1000, 2118s, Cell Signaling Technology) overnight at 4 °C. Membranes were washed with Tris-buffered saline containing 0.2% Tween 20 and incubated with horseradish peroxidase (HRP)-linked secondary antibody (1:5000, GeneTex) for 1 h. The signal was detected using Immobilon Western Chemiluminescent HRP Substrate (Merck Millipore) and visualized using a luminescent image analyzer (LAS-4000, FUJIFILM, Tokyo, Japan). Quantification was performed using ImageJ software.

### 4.9. Bacterial DNA Extraction

Fecal samples were collected into tubes with RNAlater stabilization solution (Thermo Fisher Scientific) and stored at −80 °C until analysis. Samples were thawed, and nine volumes of PBS were added and vortexed vigorously for 3–5 min. Fecal DNA was extracted from 200 μL of the mixture. Briefly, DNA extraction buffer (200 mM Tris-HCl, 80 mM EDTA, 2% SDS; pH 9.0), glass beads (diameter 0.1 mm), and phenol were added to the mixture and homogenized using a FastPrep FP 120 homogenizer (Qbiogene, Irvine, CA, USA) at 5.0 m/s for 30 s [28]. Mixtures were centrifuged at 12,000× *g* for 5 min, and the supernatants were collected for phenol–chloroform extraction and DNA precipitation. Extracted DNA was stored at −80 °C.

### 4.10. 16S rRNA Sequencing and Data Processing

PCR with specific primers for the V3–V4 regions of the 16S rRNA gene was performed to amplify the amplicons from individual samples. Amplicons were sequenced on an Illumina MiSeq platform and 300 bp paired-end reads were generated. Sequences were quality checked and clustered into operational taxonomical units with 97% similarity. The Quantitative Insights Into Microbial Ecology 2 software and the RDP Classifier Bayesian Algorithm (http://rdp.cme.msu.edu/) were used to analyze the data. Alpha diversity was analyzed by species richness and species evenness estimators, whereas beta diversity was analyzed using NMDS. The statistical significance of beta diversity was analyzed using ANOSIM. LEfSe analysis was performed to identify bacterial taxa that were differentially represented between groups. To further identify differences in the relative abundance of microbes among groups, the Mann–Whitney U-test was implemented.

### 4.11. Statistical Analysis

Data were analyzed using GraphPad Prism (version 9) and presented as the mean ± standard error of the mean. Comparison of means among groups was performed using one-way analysis of variance with Tukey’s post hoc test. Spearman’s correlation coefficient (r) was used to assess the associations between bacteria and behavioral tests, bacteria and miRNA expression, and bacteria and protein expression. Statistical significance was set at *p* < 0.05.

## 5. Conclusions

In the present study, we demonstrated that *L. plantarum* PS128 ameliorated rotenone-induced motor deficits and exerted neuroprotective effects in rotenone-induced PD-like mice. Alterations in the gut microbiota composition and dysregulation of the expression of miR-155-5p and SOCS1 were observed in PD-like mice and partially restored by PS128.

**Figure 6:**
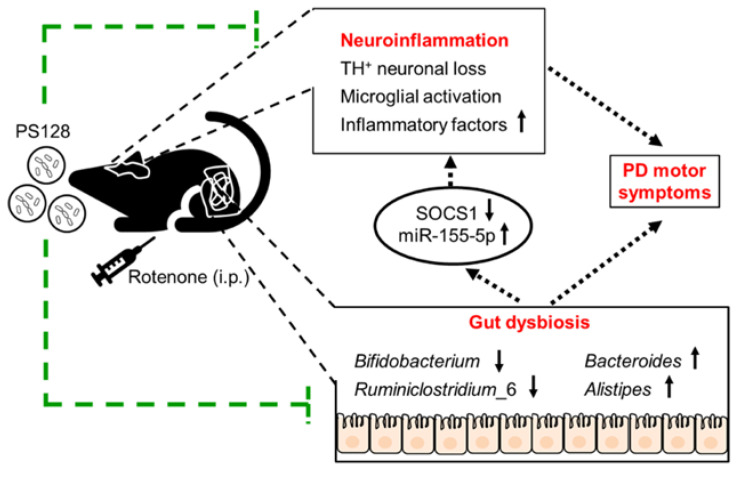
The potential mechanism by which *L. plantarum* PS128 alleviates motor symptoms in rotenone-induced PD-like mice.

Eight characteristic genera (*Bifidobacterium*, *Ruminiclostridium*_6, *Ruminococcaceae*_UCG_014, *Bacteroides*, *Adlercreutzia*, *Alistipes*, ASF356, and *Acetatifactor*) were identified to be associated with rotenone-induced PD-like symptoms. Among them, *Bifidobacterium*, *Ruminiclostridium*_6, *Bacteroides*, and *Alistipes* were observed to be significantly correlated with the expression of miR-155-5p and SOCS1 and improvement in rotenone-induced motor deficits. Thus, we suggested that by reshaping gut microbiota, PS128 regulates the miR-155-5p/SOCS1 pathway and its downstream targets and eventually confers neuroprotection, including reduced dopaminergic neuronal loss, reduced microglial activation, and suppression of inflammatory factors in the brain region of rotenone-induced PD-like mice (Figure 6). Our study proposed a protective mechanism for PS128 in rotenone-induced PD-like symptoms and provided another therapeutic approach for PD.

## Figures and Tables

**Table 1:** Effects of *L. plantarum* PS128 on the levels of microRNA (miRNA) expression in different tissues.

	Proximal colon			Midbrain			Striatum		
	Veh	Rot	PS128	Veh	Rot	PS128	Veh	Rot	PS128
miR-19b-3p	1.11 ± 0.157	1.20 ± 0.281	1.41 ± 0.187	1.01 ± 0.064	1.39 ± 0.237	1.46 ± 0.242	1.05 ± 0.124	1.05 ± 0.078	1.27 ± 0.172
miR-21-5p	1.02 ± 0.098	1.19 ± 0.089	1.62 ± 0.150 **	1.00 ± 0.096	1.74 ± 0.241	1.75 ± 0.385	1.05 ± 0.140	1.24 ± 0.191	1.16 ± 0.076
miR-34a-5p	1.03 ± 0.116	1.29 ± 0.116	1.56 ± 0.144 *	1.00 ± 0.146	1.89 ± 0.334 *	1.01 ± 0.137 ^#^	1.06 ± 0.164	1.23 ± 0.153	1.55 ± 0.283
miR-135a-5p	1.05 ± 0.153	0.99 ± 0.100	1.11 ± 0.111	1.00 ± 0.174	1.59 ± 0.198	1.58 ± 0.549	1.03 ± 0.106	1.23 ± 0.203	1.29 ± 0.260
miR-146a-5p	1.01 ± 0.060	1.44 ± 0.161	1.97 ± 0.259 **	0.99 ± 0.075	1.42 ± 0.225	1.34 ± 0.122	1.02 ± 0.071	1.18 ± 0.093	1.29 ± 0.121
miR-155-5p	1.04 ± 0.047	1.55 ± 0.112 **	0.87 ± 0.086 ^###^	0.98 ± 0.154	1.61 ± 0.107 *	0.87 ± 0.122 ^##^	1.04 ± 0.112	1.67 ± 0.145 *	0.76 ± 0.149 ^##^
miR-223-3p	1.02 ± 0.062	1.51 ± 0.121 *	0.99 ± 0.125 ^#^	1.00 ± 0.087	1.57 ± 0.369	0.65 ± 0.065^#^	1.08 ± 0.185	1.09 ± 0.168	0.93 ± 0.100

Mean fold changes of the expression of miRNAs in the proximal colon, midbrain, and striatum of rotenone-induced PD-like mice. N = 5 per group, * *p* < 0.05, ** *p* < 0.01 compared with the Veh group; # *p* < 0.05, ## *p* < 0.01, ### *p* < 0.001 compared with the Rot group.

## Data Availability

The datasets supporting this article are available in the Supplementary Materials. Further data are available from the corresponding author upon request.

## Supplementary Materials

The following supporting information can be downloaded at: https://www.mdpi.com/article/10.3390/ijms24076794/s1. Table S1. Summary of data quality. Figure S1. Differential microbiota composition of fecal specimens from Veh, Rot, and PS128 mice.
