# Supplementary material for: Neuroprotective Effects of Lactobacillus plantarum PS128 in a Mouse Model of Parkinson’s Disease: The Role of Gut Microbiota and MicroRNAs
Source: Int J Mol Sci. 2023 Apr 5;24(7):6794. doi: 10.3390/ijms24076794 (PMC10095543; doi:10.3390/ijms24076794)
Supplement: Supplementary file 1 [file ijms-24-06794-s001.zip › ijms-2290227-supplementary.pdf]

Neuroprotective Effects of *Lactobacillus plantarum* PS128 in a Mouse Model of Parkinson's Disease: The Role of Gut Microbiota and MicroRNAs

Yan Zhang Lee [1], Shih-Hsuan Cheng [2], Min-Yu Chang [2], Yu-Fen Lin [2], Chien-Chen Wu [2,*] and Ying-Chieh Tsai [1,3,*]

[1] Biomedical Industry Ph.D. Program, National Yang Ming Chiao Tung University, Taipei 11221, Taiwan
[2] Bened Biomedical Co., Ltd., Taipei 10448, Taiwan
[3] Institute of Biochemistry and Molecular Biology, National Yang Ming Chiao Tung University, Taipei 11221, Taiwan
* Correspondence: allen@benedbiomed.com (C.-C.W.); tsaiyc@ym.edu.tw (Y.-C.T.)

Supplementary Materials

Table S1. Summary of data quality.

Group	PE Reads	Q20 (%)	Q30 (%)	Average Length (bp)	GC (%)
Veh (n = 10)	65999.8	95.95; 86.36	88.43; 72.06	300.99	55.30; 55.62
Rot (n = 10)	83569.1	96.41; 87.34	89.52; 73.51	300.99	54.72; 55.07
PS128 (n = 10)	75601.3	96.29; 87.12	89.25; 73.15	300.99	54.66; 55.00

(1) The data include read 1 and read 2 from paired-end sequencing.

(2) Q20 represents an error rate of 1 in 100 bases.

(3) Q30 represents an error rate of 1 in 1000 bases.

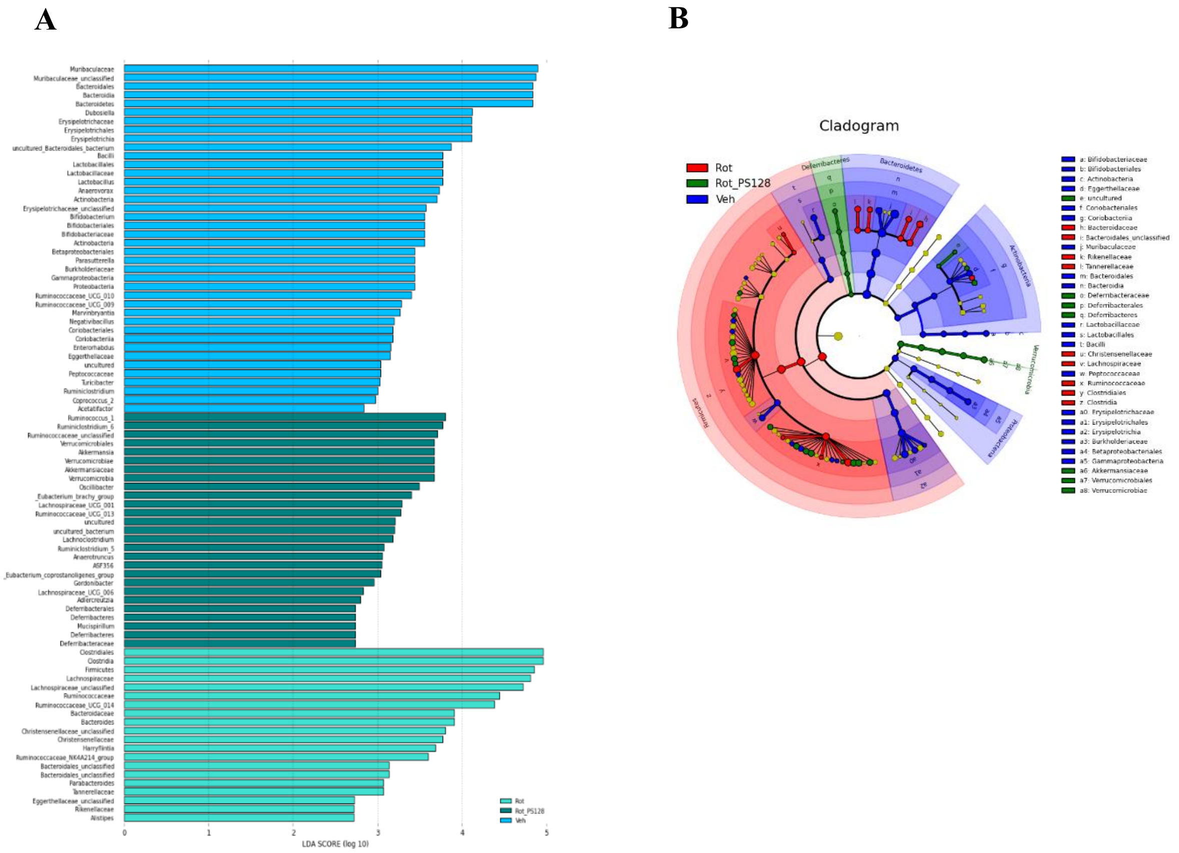

Figure S1. Differential microbiota composition of fecal specimens from Veh, Rot, and PS128 mice. (**A**) Bar plot and (**B**) cladogram of LEfSe analysis showed taxa contribution to group separation. Enriched taxa in either the Veh, Rot, or PS128 group are colored in blue, light green, and dark green, respectively. Taxa with LDA score > 2 are displayed.
